# Serum transferrin as a biomarker of hepatocyte nuclear factor 4 alpha activity and hepatocyte function in liver diseases

**DOI:** 10.1186/s12916-021-01917-6

**Published:** 2021-02-17

**Authors:** Nurdan Guldiken, Josepmaria Argemi, Berivan Gurbuz, Stephen R. Atkinson, Martin Oliverius, Petr Fila, Karim Hamesch, Tony Bruns, Joaquín Cabezas, Juan J. Lozano, Jelena Mann, Sheng Cao, Philippe Mathurin, Vijay H. Shah, Christian Trautwein, Mark R. Thursz, Ramon Bataller, Pavel Strnad

**Affiliations:** 1 Department of Internal Medicine III, University Hospital RWTH Aachen, Pauwelsstraße 30, 52074 Aachen, Germany; 2 Pittsburgh Liver Research Center, University of Pittsburgh Medical Center, Pittsburgh, PA USA; 3 Liver Unit, Clinica Universidad de Navarra, Hepatology Program, Center for Applied Medical Research, Pamplona, Spain; 4 Department of Hepatology, Imperial College London, London, UK; 5 Center of Cardiovascular Surgery and Transplantation Brno, Brno, Czech Republic; 6 Research Institute Valdecilla (Instituto de Investigación Sanitaria Valdecilla), Santander, Spain; 7 Gastroenterology and Hepatology Unit, University Hospital Marqués de Valdecilla, Santander, Spain; 8 Centro de Investigacion Biomedica en Red, Enfermedades Hepáticas y Digestivas (CIBERehd), Barcelona, Spain; 9 Newcastle Fibrosis Research Group, Biosciences Institute, Faculty of Medical Sciences, Newcastle University, Newcastle, UK; 10 Division of Gastroenterology and Hepatology, Mayo Clinic, Rochester, MN USA; 11 Hôpital Claude Huriez, Services des Maladies de l’Appareil Digestif, CHRU Lille, and Unité INSERM 995, Lille, France

**Keywords:** End-stage liver disease, Cirrhosis, Alcoholic hepatitis, Transferrin, HNF4alpha

## Abstract

**Background:**

Serum transferrin levels represent an independent predictor of mortality in patients with liver failure. Hepatocyte nuclear factor 4 alpha (HNF4α) is a master regulator of hepatocyte functions. The aim of this study was to explore whether serum transferrin reflects HNF4α activity.

**Methods:**

Factors regulating transferrin expression in alcoholic hepatitis (AH) were assessed via transcriptomic/methylomic analysis as well as chromatin immunoprecipitation coupled to DNA sequencing. The findings were corroborated in primary hepatocytes. Serum and liver samples from 40 patients with advanced liver disease of multiple etiologies were also studied.

**Results:**

In patients with advanced liver disease, serum transferrin levels correlated with hepatic *transferrin* expression (*r* = 0.51, *p* = 0.01). Immunohistochemical and biochemical tests confirmed reduced HNF4α and transferrin protein levels in individuals with cirrhosis. In AH, hepatic gene-gene correlation analysis in liver transcriptome revealed an enrichment of HNF4α signature in transferrin-correlated transcriptome while transforming growth factor beta 1 (TGFβ1), tumor necrosis factor α (TNFα), interleukin 1 beta (IL-1β), and interleukin 6 (IL-6) negatively associated with transferrin signature. A key regulatory region in transferrin promoter was hypermethylated in patients with AH. In primary hepatocytes, treatment with TGFβ1 or the HNF4α inhibitor BI6015 suppressed transferrin production, while exposure to TNFα, IL-1β, and IL-6 had no effect. The correlation between hepatic *HNF4A* and *transferrin* mRNA levels was also seen in advanced liver disease.

**Conclusions:**

Serum transferrin levels constitute a prognostic and mechanistic biomarker. Consequently, they may serve as a surrogate of impaired hepatic HNF4α signaling and liver failure.

**Supplementary Information:**

The online version contains supplementary material available at 10.1186/s12916-021-01917-6.

## Background

The liver is the largest gland of the human body, and accordingly, liver hepatocytes synthesize the majority of serum proteins [1]. Accordingly, the levels of these proteins are diminished in advanced liver disease as they reflect the loss in hepatocytes [1]. Apart from that, the protein synthesis within hepatocytes is subject to a complex regulation by liver-enriched transcription factors (LETFs). Hepatocyte nuclear factors constitute prototypical LETFs and regulate the production of multiple liver-specific proteins [2, 3]. During stress situations, various cytokines such as interleukins 1 and 6 or tumor necrosis factor α (TNFα) induce the synthesis of species known as acute-phase proteins (APPs) and diminish the production of negative APPs [1, 4]. Albumin or transferrin are characteristic negative APPs [1, 5]. Serum levels of negative APPs constitute attractive biomarkers reflecting both the hepatocellular mass and the amount of superimposed systemic stress or inflammation [1, 6]. In particular, transferrin emerges as a mortality predictor in sepsis and multiple liver disorders [1, 6, 7].

A defective hepatocyte nuclear factor 4 alpha (HNF4α)-dependent gene expression was identified as a driver of hepatocellular failure [8, 9].

Transferrin is one the most abundant serum proteins produced and secreted by the hepatocytes and serves as a chaperone capable of binding and transporting intestine-absorbed and metabolism-derived iron to other cells and tissues [10]. Its concentration in the serum is approximately 200–300 mg/dl, but decreased levels are seen during inflammation and hepatic dysfunction [1, 7, 10, 11]. Particularly low serum transferrin values occur in individuals with sepsis or acute-on-chronic liver failure [7, 12, 13]. In critically ill patients and individuals with decompensated liver cirrhosis, severe alcoholic hepatitis (AH), or acute liver failure, low transferrin levels associate with increased mortality. Notably, this association is independent of established predictive scores such as Model for End-Stage Liver Disease, markers of liver synthesis or inflammation [7, 12–17].

While transferrin represents an attractive adjunct to the existing prognostic scores, the biological mechanisms underlying its predictive power remain unclear. Here, we studied the mechanisms regulating transferrin production in individuals with advanced liver disease and AH and show that serum transferrin levels reflect the hepatic activity of HNF4α. Thus, transferrin constitutes both a prognostic and a mechanistic biomarker and an attractive surrogate for therapeutic trials aiming to preserve HNF4α signaling as a tool to prevent the development of liver failure.

## Methods

### Human serum and liver specimens for biochemical analysis (cohort i)

Forty liver samples from patients who underwent a liver biopsy at the Universities of Aachen and Ulm between the years 2006 and 2018 were analyzed. The diagnosis of HCV was confirmed by a positive HCV RNA test, and all patients were treatment-naive. The diagnosis of ALD/NAFLD was based on laboratory parameters, histological findings, personal interview, and an exclusion of other liver disease etiologies including viral infection, hereditary hemochromatosis, and autoimmune liver disease. NAFLD patients had characteristic histological findings without significant alcohol consumption (Table 1). Among them, a matched serum pair was available for 23 samples that were used for correlation analysis. Four liver samples from surrounding, non-affected tissue of liver metastases from patients without known chronic liver disease had been used as controls (Table 1). Staging of fibrosis was done on 4-μm-thick liver sections using Desmet (HCV, controls) and Kleiner (ALD, NAFLD) scores. Publicly available data on transferrin levels in liver disease were obtained from GEO database and in silico analysis was performed on the GSE103580 dataset in 13 patients with alcoholic hepatitis [18]. The study was conducted in compliance with the Declaration of Helsinki (Hong Kong amendment) and Good Clinical Practice (European guidelines) as reflected in an approval by the local ethics committees of the participating centers. All participants, or their legally appointed representatives, provided written informed consent.

**Table 1:** Patient’s characteristics (cohort i)

Sample cohort	M/F	Age*	Fibrosis stage*
*Control*^*+*^	3/1	61.2 ± 16.6	0 ± 0
*ALD*	10/4	53.4 ± 10.4	3.6 ± 0.5
*NAFLD/NASH*	3/5	58.1 ± 11.6	3.6 ± 0.5
*HCV*	7/3	56.9 ± 6.5	4 ± 0
*Other etiologies*^*#*^	2/6	52.4 ± 15.8	4 ± 0

*ALD* alcoholic liver disease, *HCV* chronic hepatitis C infection, *NAFLD* non-alcoholic fatty liver disease, *NASH* non-alcoholic steatohepatitis

^#^Other etiologies include one patient with chronic hepatitis B and ALD, one with autoimmune hepatitis, one with cryptogenic cirrhosis, and one with biliary cirrhosis

^+^Control samples were used for western blotting and staining

*Data are expressed as mean ± SD

### Transcriptomic analysis in human liver specimens (cohort ii)

RNA-seq was performed on liver tissue biopsies obtained from patients with early alcoholic steatohepatitis (*n* = 12), non-severe AH (*n* = 11), severe AH (*n* = 18), explants from patients transplanted with severe AH (*n* = 9), and normal background liver specimens from patients resected for benign lesions (*n* = 10), from the *InTEAM Consortium - Alcoholic Hepatitis Liver RNA Sequencing* study, sponsored by the National Institute of Alcohol Abuse and Alcoholism (NIAAA, USA)*.* The study details and sequencing data can be found in the Database of Genotypes and Phenotypes (dbGAP, phs001807.v1.p1) of the National Institutes of Health (NIH, USA). The basic clinical and laboratory data of the patients included in this study, the methods used to extract RNA and perform deep RNA sequencing, and the bioinformatic pipelines used to determine transcript counts have been described elsewhere [9]. In order to uncover the transferrin co-expressed transcriptome, the Kendall rank correlation coefficient was calculated. For pathway analysis, directly and reversely correlated genes with *p* value inferior to 10^−12^ were selected. The Kendall coefficient ranges for selected genes was + 0.76 to + 0.91 for the directly correlated (*n* = 309 genes) and − 0.76 to − 0.84 for the reversely correlated (*n* = 94 genes). Ingenuity Pathway Analysis (IPA, Qiagen Inc., version 2017) was used to uncover main upstream regulators.

### Genomic DNA methylome analysis (cohort ii)

Liver DNA methylation studies have been described previously [9]. Briefly, genomic DNA (gDNA) was extracted from flash-frozen liver tissue with PureLink Genomic DNA Mini Kit (Thermo) and quantified using Nanodrop (Thermo). One microgram of isolated gDNA was bisulfite converted, denatured, fragmented, and hybridized to Infinium Methylation Bead Chip, following the manufacturer’s protocol (Infinium MethylationEPIC kit, Illumina). BeadChips were imaged using an Illumina Scan System, and intensity was determined by the iScan Control Software (Illumina). Sample intensities were normalized using functional normalization from the *minfi* package (v1.24.0) [19]. Probes failing a detection *p* value threshold (0.01) in at least 50% of samples were removed, as were probes identified as containing a SNP with a MAF > 0.05. Differentially methylated probes were identified by applying *limma* (v3.34.3) contrasts to *M* values (absolute change in beta value > 0.1, FDR-corrected *p* value < 0.05) [20]. Differentially methylated regions were identified using DMRcate (v1.14.0) [21] setting a threshold of absolute change in beta value in > 0.1 and of Stouffer’s value in < 0.05. The package gviz (v 1.28.0) [22] was used to visualize *transferrin* genomic region, including methylation beta values. The information downloaded from the University of California Santa Cruz (UCSC) server of the Human Genome Sequencing Consortium included HepG2 HNF4A ChIP-seq BigWig data (Sample GSM803460 of series GSE32465) and DNAse I hypersensitive sites Bed data (wgEncodeRegDnaseClusteredV3.bed, UW and Duke ENCODE data).

### Chromatin immunoprecipitation and DNA sequencing (ChIP-seq) of histone marks (cohort ii)

ChIP-seq was performed in Mayo Epigenomics Development Laboratory (EDL) [23] with the liver tissue from 5 controls and 7 severe AH explants (provided by University of Lille, France). It was carried out for four histone modifications, using antibodies against histone H3 Lysine 27 acetylation (H3K27ac, Cell Signaling #8173), histone H3 Lysine 27 tri-methylation (H3K27me3, Cell Signaling #9733), histone H3 Lysine 4 mono-methylation (H3K4me1, EDL, Mayo Clinic, Lot#1), and histone H3 Lysine 4 tri-methylation (H3K4me3, EDL, Mayo Clinic, Lot#1). For the next-generation sequencing, ChIP-seq libraries were prepared from 10 ng of ChIP and input DNAs with the Ovation Ultralow DR Multiplex System (NuGEN). The ChIP-seq libraries were sequenced to 51 base pairs from both ends using the Illumina HiSeq 2000 in the Mayo Clinic Medical Genomics Core. Data were analyzed by the HiChIP pipeline [24]. Briefly, reads were aligned to the hg19 genome assembly using BWA and visualized using the Integrative Genomics Viewer (IGV). Mapped reads were post-processed to remove duplicates and pairs of reads mapping to multiple locations. The MACS2 and Sicer algorithm was used for peak calling in relation to the input DNA. In this study IGV was then used to visualize the peak changes on *transferrin* genomic region in this study.

### Biochemical analysis (cohort i)

Four control and 6 cirrhotic samples were used for biochemical analysis. Tissue lysates were prepared in homogenization buffer containing 3% sodium dodecyl sulphate (SDS) and were diluted with 4× reducing Laemmli buffer afterwards. Proteins were separated via 10% SDS polyacrylamide gel electrophoresis and transferred to PVDF membranes (GE Healthcare/Amersham Biosciences, Germany) for immunoblotting. After incubation with the primary and horse radish peroxidase (HRP)-coupled secondary antibodies, the resulting HRP signal was detected with an ECL Detection kit (GE Healthcare/Amersham Biosciences, UK). The following antibodies were used: human anti-transferrin (Sigma; HPA005692, Stockholm, Sweden), mouse anti-transferrin (Abcam; Ab82411), anti-HNF4alpha (Sigma; HPA004712, Bromma Sweden), and anti-GAPDH (Novus; NB300-221, Nordenstadt, Germany).

### Tissue stainings (cohort i)

For immunohistochemistry, 3–4-μm-thick liver sections were deparaffinized and rehydrated, and a citrate-based antigen retrieval was performed (Vector H-3300, Vector Laboratories, Petersborough, UK) for 30 min, as recommended by the supplier. The immunodetection was carried out with anti-transferrin as a primary (11,000 dilution; Sigma; HPA005692, Stockholm, Sweden) and a biotin-conjugated anti-rabbit secondary antibody (Vector BA1000). The signal was visualized with peroxidase-labeled streptavidin and the 3,3′-diaminobenzidine as a substrate (Vector SK4100).

### Measurement of serum parameters (cohort i)

Serum transferrin (turbidimetry), iron, and ferritin levels were measured with routine assays using the Cobas 8000 system (Roche Diagnostics, Mannheim, Germany) available at the Clinical Chemistry Department of University Hospital Aachen. Serum apolipoprotein B (APOB; R&D Systems DAPB00, Minneapolis, MN, USA), human tranthyretin (Prealbumin; Avivasysbio, OKIA00081, San Diego, CA, USA), and complement C3 (Abcam, ab108822, Berlin, Germany) concentrations were measured by using commercial sandwich ELISAs.

### Primary hepatocyte cell culture

Primary hepatocytes were isolated from 3- to 4-month-old C57BL/6 mice as described previously [25]. Primary hepatocytes were kept in culture medium (DMEM; PAN Biotech, Aidenbach, Germany) supplemented with 10% FBS, 1% penicillin-streptomycin, and l-glutamine (PAN Biotech). After overnight incubation, the medium was replaced by William’s E medium (PAN Biotech), and cells were treated for 6 h with IL-1β (20 ng/ml; Peprotech, Hamburg, Germany), IL-6 (40 ng/ml; Sigma, St. Louis, MO, USA), TNFα (30 ng/ml; Peprotech), and the HNF4α antagonist BI6015 (50 μM; Millipore, Burlington, MA, USA) or for 24 h with TGFβ1 (10 ng/ml; R&D Systems, Minneapolis, MI, USA). Alternatively, the cells were transfected with the siRNA against HNF4α (60 pmol; Thermo Fisher Scientific ID: 158081 and 158082, Germany) according to the manufacturer’s protocol (Lipofectamine® RNAiMAX™ Transfection Reagent, Thermo Fisher Scientific, Germany) and cultured for 48 h. RNA was isolated via RNeasy tissue mini isolation kit (Qiagen, Hilden, Germany). The RNA samples were translated to cDNA with the M-MLV reverse transcriptase kit (Promega, Madison, WI, USA) and random hexamers (Thermo Scientific, Waltham, MA, USA). The relative expression of genes of interest was determined using specific primers (Additional file 1: Table S1). The mouse ribosomal gene L7 was used as an internal loading control.

### Data processing and statistical analysis

Based on the results of a normality test, continuous variables were compared by an unpaired *t* test. Correlations between clinical variables and serum proteins were tested by Spearman’s rank correlation test. Nominal two-sided *p* values were reported for all tests and were considered to be statistically significant when *p* < 0.05 unless mentioned otherwise. Statistical analyses were performed, and graphs were created with GraphPad Prism (GraphPad, La Jolla, CA, USA).

## Results

### Patients with advanced liver disease display decreased hepatic transferrin expression

**Fig. 1:**
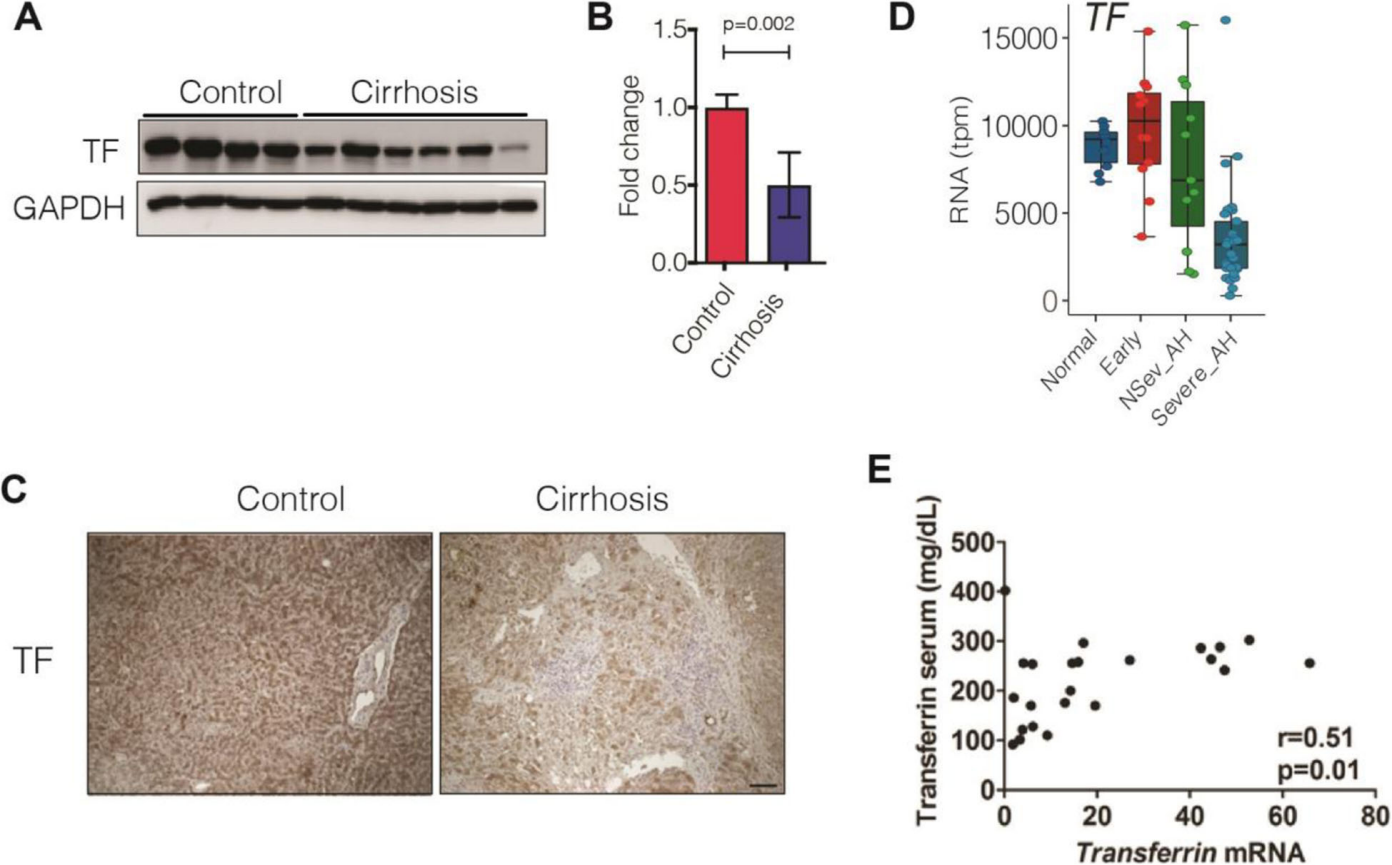
Transferrin serum levels reflect the decreased transferrin expression in advanced liver disease. **a**, **b** Hepatic transferrin protein levels were determined by immunoblotting in cirrhotic patients (cirrhosis) and individuals without a significant liver disease (control). GAPDH was used as a loading control. The relative band intensity was quantified with the ImageJ software (cohort i). **c** Immunohistochemical staining of liver sections with an antibody against transferrin. Scale bar = 200 μm (cohort i). **d** Normalized transcripts per million base pairs (tpm) of the Transferrin gene in patients with alcohol-related liver disease and normal livers. The samples were obtained from biopsies from patients with normal background liver (“Normal,” *N* = 10), patients with early or silent alcohol-related steatohepatitis (“Early,” *N* = 12), and patients with alcoholic hepatitis (AH), at different disease stages: non-severe (NSev_AH, *N* = 11) and severe (Severe_AH, *N* = 27, including biopsies *N* = 18 and explants *N* = 9) (cohort ii). **e** Spearman’s correlation coefficient reveals the relationship between serum transferrin levels and relative hepatic transferrin mRNA expression in 23 patients with advanced liver disease and available matched serum-mRNA sample pairs (cohort i)

To delineate the factors underlying the predictive usefulness of transferrin in severe liver disease, we compared the hepatic expression of *transferrin* and transferrin serum levels in 23 patients with advanced liver disease and available matched mRNA-serum sample pairs (Table 1) (cohort i). Immunoblotting revealed decreased transferrin protein levels in cirrhotic livers (Fig. 1a, b). Accordingly, immunohistochemistry manifested a strong, homogeneous hepatocellular staining in control livers, whereas a patchy, diminished signal was seen in cirrhotic patients (Fig. 1c) (cohort i). In individuals with different stages of AH (cohort ii), a progredient decrease in normalized transferrin gene transcripts was seen at more severe stages (Fig. 1d). Spearman’s correlation showed a moderate correlation between the serum transferrin levels and hepatic mRNA expression in individuals with advanced liver fibrosis and available matched serum-mRNA sample pairs (Fig. 1e) (cohort i). Notably, serum transferrin levels did not correlate with serum levels of other established hepatocellular products such as alpha1-antitrypsin, ceruloplasmin, albumin, or apolipoprotein B (not shown). On the other hand, in cohort (i), we observed a moderate negative correlation between serum ferritin and transferrin levels, while no correlation was seen between serum transferrin and serum iron levels (Additional file 1: Fig. S1).

### HNF4α regulates hepatocellular transferrin production

**Fig. 2:**
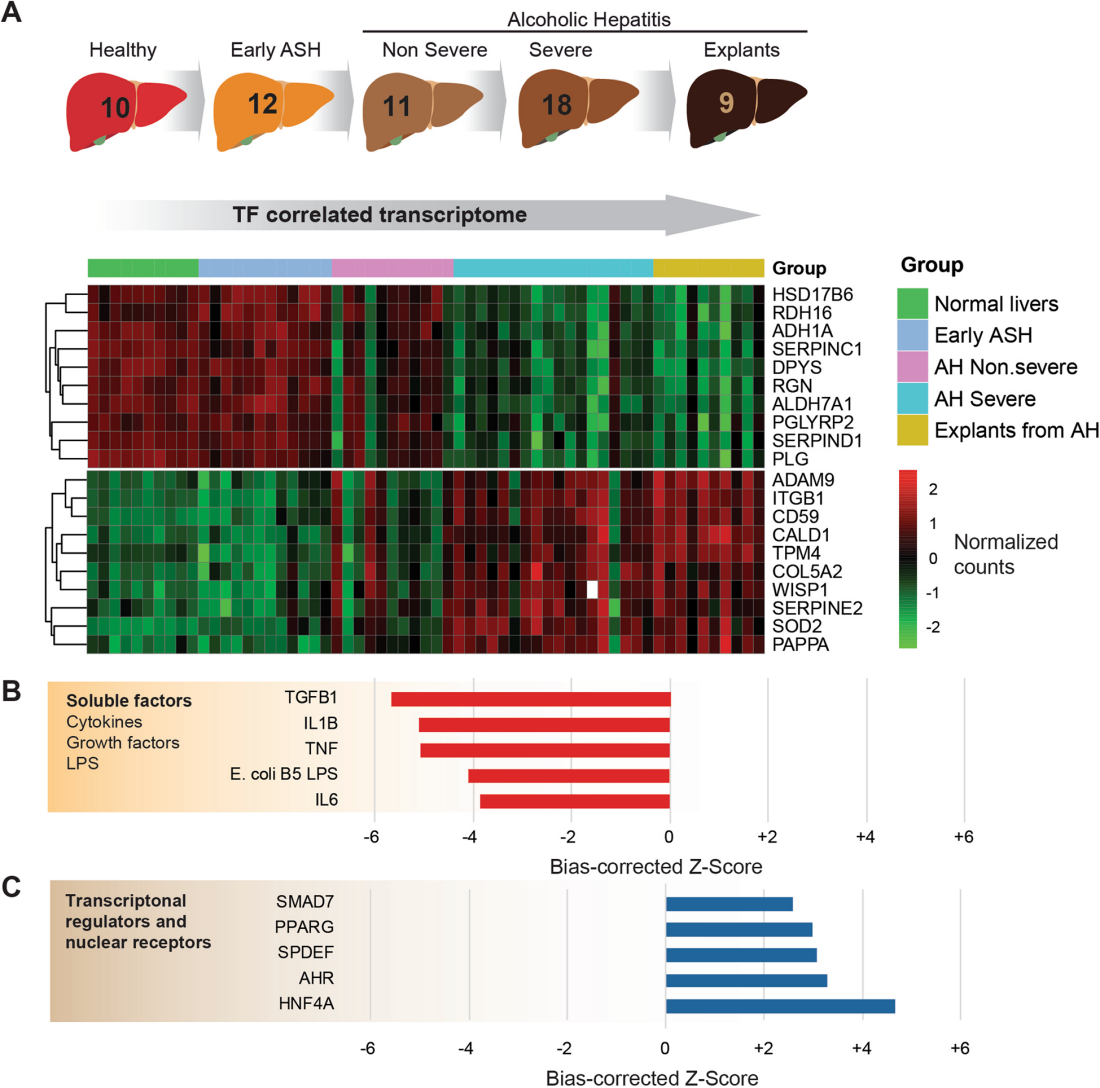
RNA-seq analysis of liver samples from patients with and without alcoholic hepatitis (AH). **a** RNA-seq analysis was carried out on liver samples from liver-healthy individuals as well as patients with different manifestations of alcoholic liver disease such as alcoholic steatohepatitis (ASH) and various stages of AH (cohort ii). A heatmap displays the normalized counts of the 10 genes most directly correlated (**b**) and most reversely correlated (**c**) with *transferrin* expression, by means of the Kendall test (cohort ii)

To determine the factors responsible for altered hepatic *transferrin* expression, we analyzed RNA-seq data from healthy individuals as well as patients with different stages of alcohol-related liver disease (ALD). This cohort comprised the full spectrum of ALD, from early asymptomatic disease in heavy drinkers to patients with alcoholic hepatitis (Figs. 1d and 2a) (cohort ii). The expression of TF in the liver of patients with AH was significantly reduced in patients with severe AH (Fig. 1d). We then performed a gene-gene correlation analysis of TF expression with the whole transcriptome, stratifying by disease severity (Fig. 2a). The most correlated genes were then used to infer transcription factor footprint using Ingenuity Pathway Analysis. The analysis of upstream regulators revealed HNF4α and TGFβ1 as the factors that are most tightly linked to the transferrin-correlated transcriptome (Fig. 2b, c) (cohort ii). This result suggests that *transferrin* expression could be a bona fide marker of hepatic HNF4α downregulation. The established drivers of acute phase response, i.e., TNFα, IL-1β, and IL-6 also displayed a strong negative correlation with *transferrin* mRNA in our upstream regulation analysis (Fig. 2b). Since these analyses were made using whole liver RNA, the impact of infiltrating non-parenchymal cells on these transcriptomic footprints cannot be excluded.

### Epigenetic and molecular regulation of hepatic transferrin expression

**Fig. 3:**
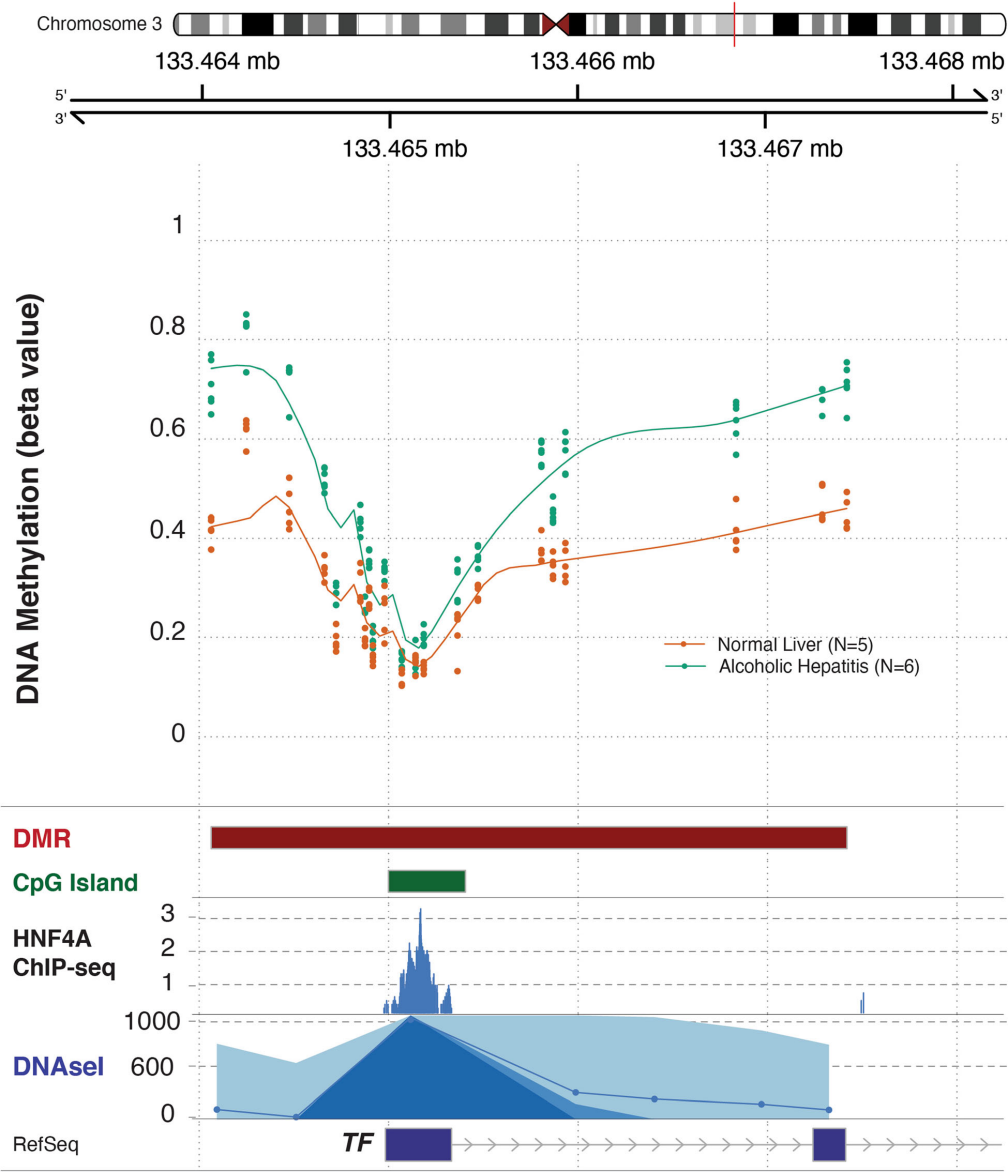
Methylomic analysis of the human transferrin gene. DNA methylation data was obtained from 5 control livers and 6 livers from patients with severe alcoholic hepatitis. A differentially methylated region (DMR) including 19 CpG around an annotated CpG island was unbiasedly detected by using Illumina EPIC Infinium methylome chip in the transferrin promoter. The methylation beta values and the length of the DMR and the CpG Island are shown. The genomic region of *transferrin* gene promoter, located in chromosome 3, was visualized by using the gviz package, with the addition of the following ENCODE genomic tracks: HNF4A ChIP seq and DNAse I hypersensitive, both in HepG2 cells (cohort ii)

**Fig. 4:**
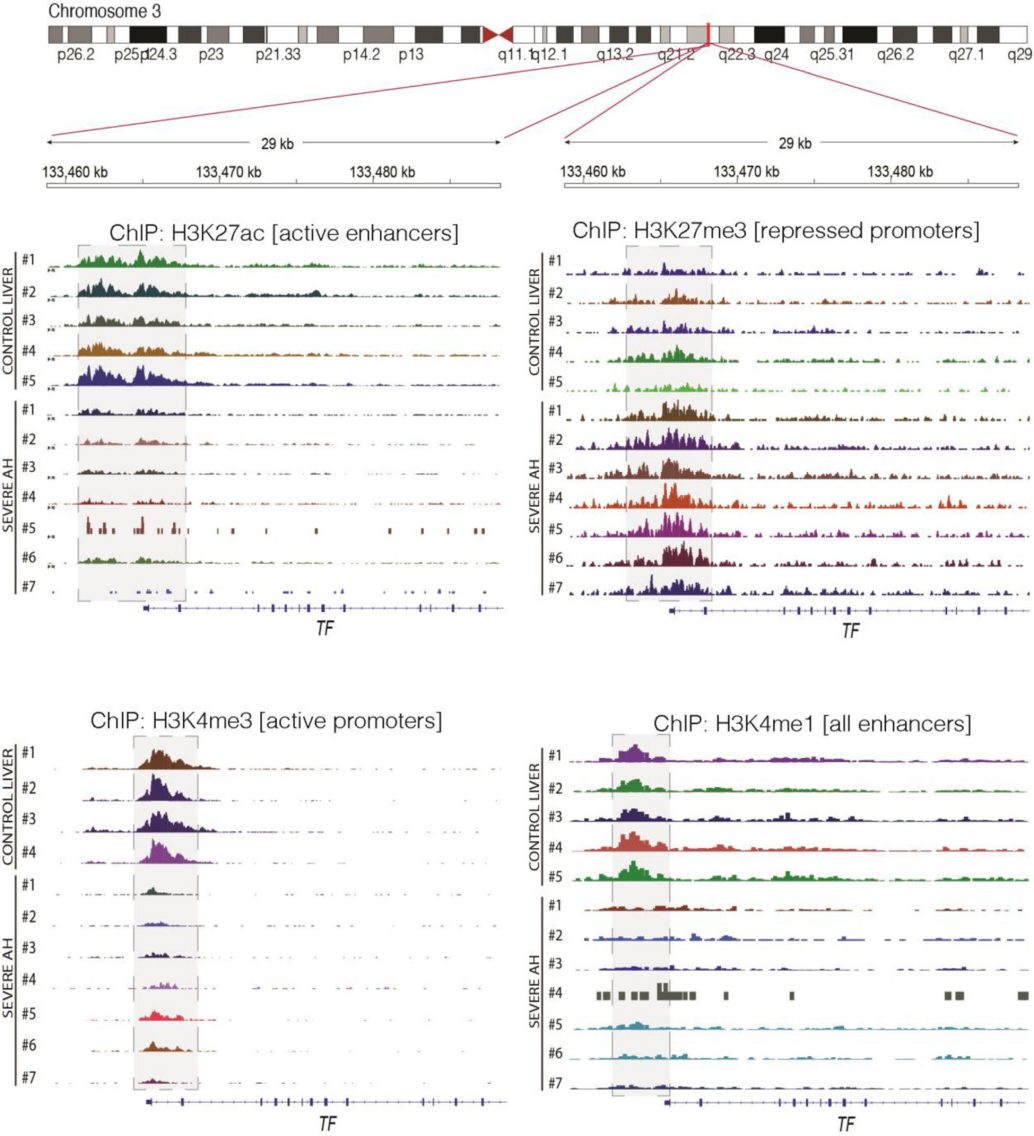
Visualization of histone modifications by chromatin immunoprecipitation and DNA sequencing (ChIP-seq) in the human transferrin genomic region. ChIP-seq data was obtained from explants from 5 control livers and 7 livers of patients with severe non-responder alcoholic hepatitis. The genomic region around the transferrin promoter was visualized through the Integrative Genome Viewer (IGV). Histone 3 lysine 4 (H3K4) mono- and tri-methylation as well as H3K27 acetylation and tri-methylation are shown. The decrease in H3K27ac signal indicated the lack of activity in transferrin enhancer and promoter regions, while the decrease of H3K4me3 and increase of H3K27me3 suggested the absence of activity and repressive chromatin configuration, respectively, in the region around the transcription start site (TSS). The light gray areas indicate the regions where these changes were seen (cohort ii)

**Fig. 5:**
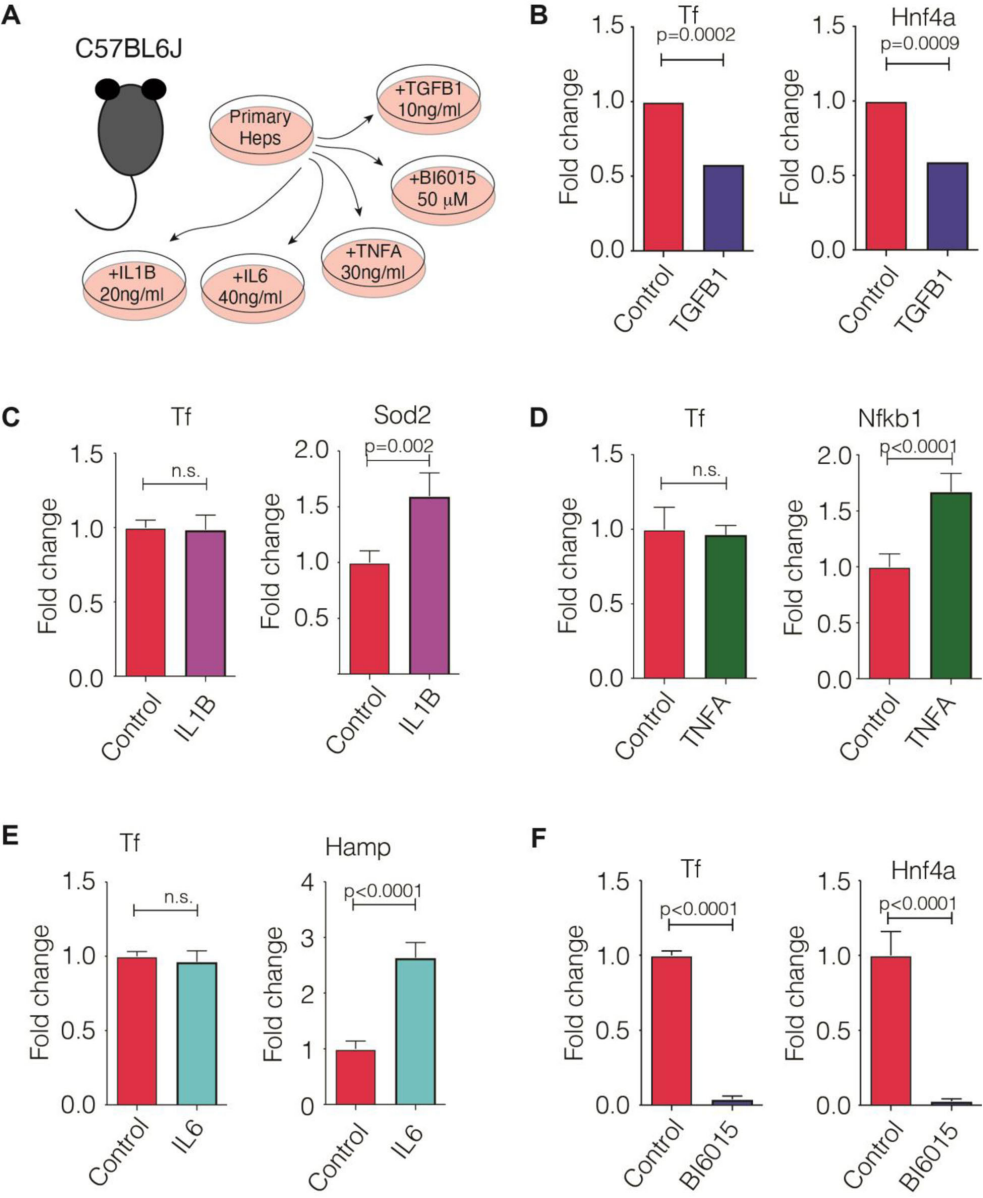
Hepatic transferrin production is regulated by the hepatocyte nuclear factor 4α (HNF4α) axis. **a** *Transferrin* mRNA expression was determined in primary hepatocytes that were treated with a carrier only (control) or subjected to TGF β1 (**b**), IL-1β (**c**), TNFα (**d**), IL-6 (**e**), or the HNF4α antagonist BI6015 (**f**). The expression of *Hnf4α*, *Sod2*, *Nfkb1*, and *Hamp* were used as positive controls. In **b**, **d**, and **f**, 5 controls and 6 treated samples were used, whereas 4 controls and 4 treated samples were assessed in **c** and **e**. The average expression in control cells was arbitrarily set as 1 and all other levels represent a ratio. Mouse *L7* (ribosomal) gene was used as an internal control

To further understand the regulation of transferrin in patients with AH, we analyzed the *transferrin* genomic region using liver methylomic data as well as chromatin immunoprecipitation coupled to DNA sequencing data (ChIP-Seq) from a previous study in patients with different stages of alcoholic liver disease (Figs. 3 and 4) [9] (cohort ii). We compared these genomic regions with different epigenetic marks and HNF4A ChIP-seq available in ENCODE. The methylome analysis revealed a differentially methylated region in a regulatory region of the *transferrin* promoter, which was bound by HNF4α. Patients with AH had higher levels of methylation in different regulatory CpG islands in this region, indicating a possible cause of *transferrin* downregulation (Fig. 3). In a different set of AH patients, ChIP-Seq showed increased levels of H3K27me3 mark and a decrease on H3K4me3 and H3K27ac marks in the same region, indicating that the chromatin conformation promotes repression of *transferrin* gene expression in these patients (Fig. 4). To test whether these associations imply a direct regulation, we used primary mouse hepatocytes (Fig. 5a). In these, TGFβ1 treatment markedly suppressed *transferrin* expression (Fig. 5b) whereas IL-1β, TNFα, and IL-6 treatment did not have a significant effect (Fig. 5c–e). Treatment with the HNF4α antagonist BI6015 resulted in an almost complete suppression of *transferrin* expression (Fig. 5f), while no significant alterations were seen in HNF4γ levels that are known to be affected by high doses of BI6015 (not shown). RT-PCR for HNF4A, which was used to confirm the efficacy of the described treatments, revealed the suppression of HNF4α expression in both the antagonist- and TGFβ1-treated hepatocytes (Fig. 5b, f). Finally, a treatment with HNF4A siRNA resulted in an almost complete suppression of *HNF4A* mRNA levels and significantly reduced transferrin protein levels (Additional file 1: Fig. S2).

### Transferrin is superior to other genes as a surrogate marker of HNF4α activity

Finally, to further corroborate the correlation between *HNF4A* and *transferrin* expression, we analyzed publicly available data on 13 patients with AH (GSE103580) [18]. In these individuals, we observed a strong association between hepatic *transferrin* and *HNF4A* levels (*r* = 0.70 *p* = 0.007; Additional file 1: Fig. S3A)*.* A comparably strong correlation was seen in 40 patients with advanced liver disease (*r* = 0.59 *p* < 0.0001; Additional file 1: Fig. S3B). These data strongly suggest that transferrin decrease reflects an impaired HNF4α axis. In line with that, immunoblotting demonstrated decreased HNF4α protein levels in cirrhotic vs. control livers (Additional file 1: Fig. S3C).

To identify additional markers of impaired HNF4α signaling, we assessed three established HNF4α target genes, i.e., apolipoprotein B (ApoB), transthyretin (TTR), and complement C3 (C3). Spearman’s correlation coefficient revealed a moderate correlation between the mRNA levels of HNF4A and its targets ApoB and C3 but no significant correlation with TTR (Additional file 1: Fig. S4). Moreover, in none of the analyzed species, a significant correlation between serum and hepatic mRNA was detected (Additional file 1: Fig. S4). Notably, similar results were seen when only subjects with ALD/NAFLD were included in the analysis (not shown). Collectively, our data indicate that transferrin may constitute an attractive serological marker reflecting the hepatic HNF4α signaling.

## Discussion

In our study, we systematically analyzed the factors regulating the serum transferrin levels and demonstrated that serum transferrin levels correlate with hepatic transferrin production and that hepatic transferrin production is not only directly regulated by, but also reflects, the activity of HNF4α signaling. The former findings are well in line with the study from Potter et al., who analyzed transferrin synthesis and serum levels in individuals with alcoholic liver disease [11]. The finding that *transferrin* mRNA levels strongly correlate with hepatic HNF4α levels is compatible with the data from liver-specific HNF4α knockout mice, which displayed a deranged iron metabolism and a diminished transferrin expression [26]. Notably, transferrin expression is not altered after exposure to TNFα, IL-1β, and IL-6 that constitute the major regulators of hepatocellular acute phase response [1, 6]. While we focused on AH and advanced liver disease, the decreased transferrin levels seen in individuals with acute liver failure and in critically ill patients [7, 14] suggest that similar mechanisms may apply to multiple different stress situations. This is of particular interest, since no correlation between hepatic expression and serum protein levels was seen for three other well-known HNF4α targets. However, our study has also important limitations. First, it primarily focused on alcoholic liver disease, and further studies are needed to determine to what extent our findings apply to other disease etiologies. Additionally, the impact of hepatic iron load on serum transferrin levels needs to be assessed.

Our data demonstrating that transferrin levels indicate the activity of the HNF4α axis is of obvious relevance. HNF4α constitutes a major liver-enriched transcription factor, that is responsible for the production of multiple mature hepatocyte-specific genes [26, 27]. Moreover, HNF4α activity is diminished in multiple liver disorders including AH and decompensated liver cirrhosis and is likely crucial for their pathogenesis [2, 9]. In line with that, a forced re-expression of HNF4α rapidly reversed fatal liver failure in CCl4-treated rats [28]. In agreement with our data, the downregulation of HNF4α signaling in AH occurs via TGFβ signaling and is thought to be mediated through DNA methylation and chromatin remodeling [9, 29]. On the other hand, the regulation of HNF4α signaling and the role of methylation remain to be systematically analyzed. Notably, a smaller study reported a correlation between serum levels of transferrin and TGFβ in patients admitted to an internal medicine department [30]. While it is tempting to speculate that decreased transferrin levels are induced via TGF β-induced, epigenetic changes in transferrin production, further studies are needed to corroborate this hypothesis.

The observation that serum transferrin is both a prognostic and a mechanistic biomarker may have direct medical implications. As such, transferrin levels can be used for patient stratification and treatment allocation in targeted clinical trials aiming to enhance HNF4α signaling. In that respect, the FDA-approved PPARγ agonist rosiglitazone was suggested as a potential candidate for the treatment of liver failure [9]. Transferrin represents an attractive surrogate of mortality in such trials. Second, lowered transferrin levels may not be only an indicator of imminent liver failure, but also a promising candidate for plasma-based therapeutic interventions. While administration of transferrin yielded promising results in various clinical conditions such as atransferrinemia and iron overload or in animal models of β-thalassemia [31, 32], it was never tested in the context of liver disease. This approach might be particularly attractive given the emerging therapeutic value of plasma exchange and/or the well-documented usefulness of plasma products such as albumin in the treatment of both acute and chronic liver failure [33, 34].

## Conclusions

Our study reports that transferrin is a novel serological surrogate of impaired HNF4α axis and as such might be useful both for prognostic evaluation as well as to direct and stratify patients for novel therapeutic approaches. Prospective studies are warranted to corroborate the relationship between HNF4α and transferrin levels in several forms of liver failure, i.e., acute liver failure, acute-on-chronic liver failure, and AH.

## Supplementary Information

**Additional file 1 **: **Figure S1-S4**. **Figure S1** Correlation between serum parameters of iron metabolism in samples from cohort (i). **Figure S2** Hepatic transferrin production is regulated by the hepatocyte nuclear factor 4α (HNF4α) axis. **Figure S3** *Transferrin* expression correlates with *HNF4α* expression in liver disease. **Figure S4** Correlation of selected HNF4α targets with the hepatic HNF4α expression in patients with advanced liver disease (cohort i). **Table S1** Primer sequences used for experiments in primary hepatocyte culture.

## Data Availability

The datasets used and analyzed during the current study are available from the corresponding author on reasonable request.

## Abbreviations

AH: Alcoholic hepatitis
ALD: Alcohol-related liver disease
APOB: Apolipoprotein B
APPs: Acute-phase proteins
ASH: Alcoholic steatohepatitis
C3: Complement C3
CCl4: Carbon tetrachloride
ChIP-Seq: Chromatin immunoprecipitation coupled to DNA sequencing data
CRP: C-reactive protein
dbGAP: Database of Genotypes and Phenotypes
GAPDH: Glyceraldehyde-3-phosphate dehydrogenase
HAMP: Hepcidin antimicrobial peptide
HCV: Chronic hepatitis C infection
HNF4α: Hepatocyte nuclear factor 4 alpha
IL-1β: Interleukin 1 beta
IL-6: Interleukin 6
L7: Large ribosomal subunit
NAFLD: Non-alcoholic fatty liver disease
NASH: Non-alcoholic steatohepatitis
NFKB1: Nuclear factor of kappa light polypeptide gene
PPARγ: Peroxisome proliferator-activated receptor γ
RPLPO: Ribosomal protein lateral stalk subunit P0
SOD2: Superoxide dismutase 2
TF: Transferrin
TGF β1: Transforming growth factor beta 1
TNFα: Tumor necrosis factor alpha
TTR: Transthyretin
