# Supplementary material for: Serum transferrin as a biomarker of hepatocyte nuclear factor 4 alpha activity and hepatocyte function in liver diseases
Source: BMC Med. 2021 Feb 17;19:39. doi: 10.1186/s12916-021-01917-6 (PMC7887823; doi:10.1186/s12916-021-01917-6)
Supplement: Supplementary file 1 — Additional file 1 : Figure S1-S4. Figure S1 Correlation between serum parameters of iron metabolism in samples from cohort (i). Figure S2 Hepatic transferrin production is regulated by the hepatocyte nuclear factor 4α (HNF4α) axis. Figure S3 Transferrin expression correlates with HNF4α expression in liver disease. Figure S4 Correlation of selected HNF4α targets with the hepatic HNF4α expression in patients with advanced liver disease (cohort i). Table S1 Primer sequences used for experiments in primary hepatocyte culture. [file 12916_2021_1917_MOESM1_ESM.docx]

**Additional file 1 of Guldiken N, Argemi J- “***Serum Transferrin as a Biomarker of Hepatocyte Nuclear Factor 4 Alpha Activity and Hepatocyte Function in Liver Diseases***”**

**Additional file 1: Fig. S1. Correlation between serum parameters of iron metabolism in samples from** **cohort (i)**. Spearman´s correlation coefficient quantifies the relationship between the serum levels of transferrin on x-axis and iron **(A)**, transferrin saturation **(B)** or ferritin levels **(C)** on y-axis.

**
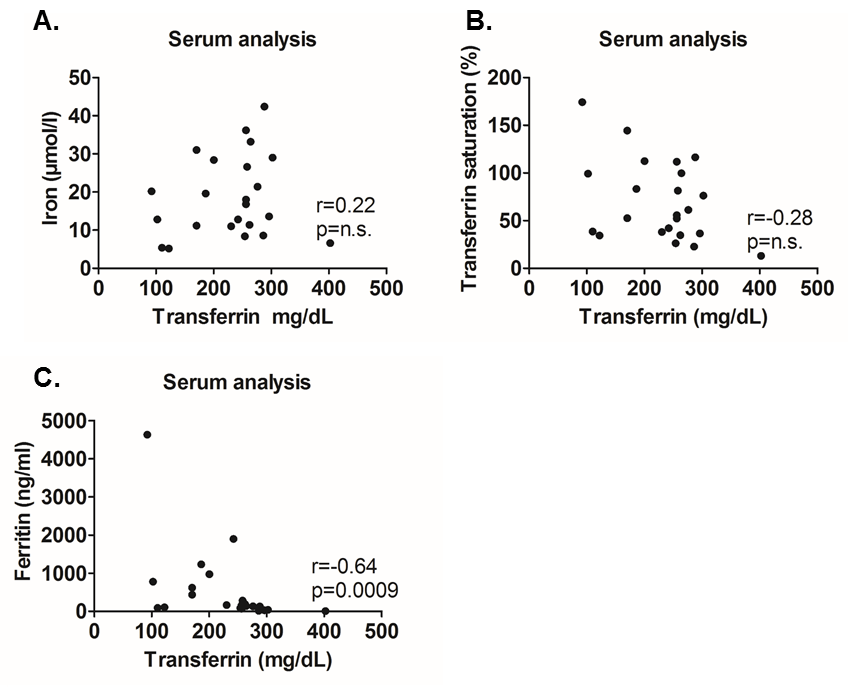
**

**Additional file 1: Fig. S2. Hepatic transferrin production is regulated by the hepatocyte nuclear factor 4α (HNF4α) axis.** *HNF4A* mRNA expression **(A)** and transferrin protein levels **(B)** were determined in primary hepatocytes treated with HNF4A siRNAs or non-targeting siRNA (n=3). Mouse *L7* (ribosomal) gene **(A)** and GAPDH **(B)** were used as loading controls. The average expression in control cells was arbitrarily set as 1 and all other levels represent a ratio.


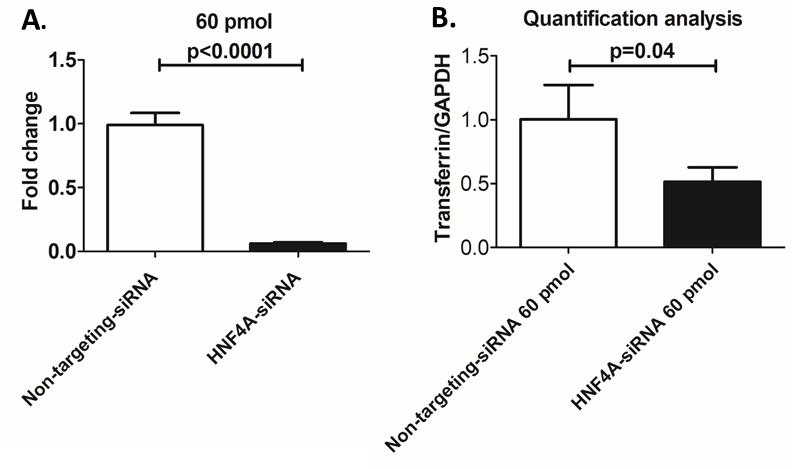


**Additional file 1: Fig. S3. *Transferrin* expression correlates with *HNF4****α* **expression in liver disease.** **(A,B)** Spearman´s correlation between the hepatic expression of *transferrin* and *HNF4A* in 13 patients with alcoholic hepatitis (GSE103580) **(A)** and in 40 patients with advanced liver disease **(cohort i)** **(B**; Table 1). Expression was normalized to the housekeeping gene *RPLPO*. **(C)** The indicated proteins were determined by immunoblotting in cirrhotic patients (cirrhosis) and individuals without a significant liver disease (control) **(cohort i)** and the relative HNF4α band intensity (compared to GAPDH) was quantified using ImageJ.


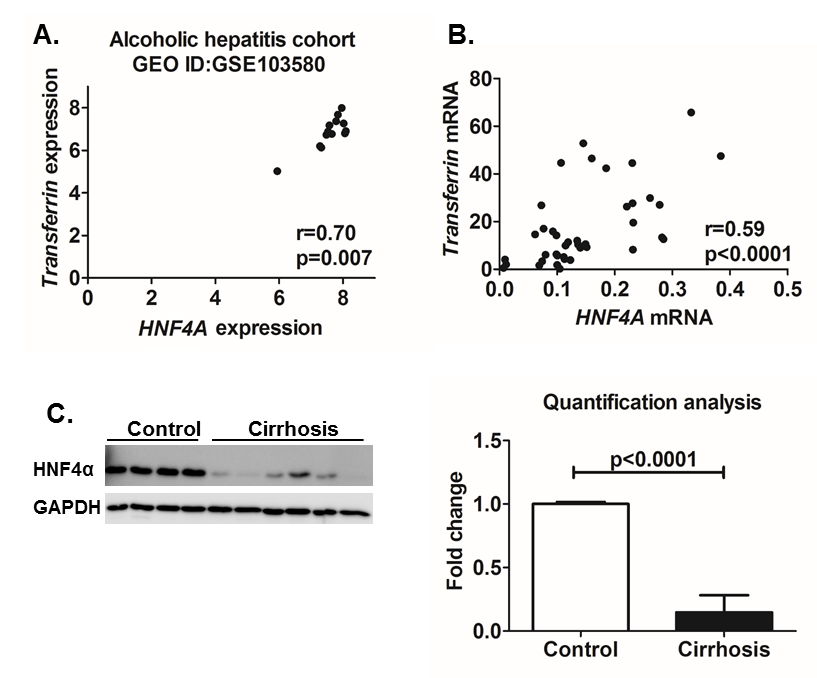


**Additional file 1: Fig. S4. Correlation of selected HNF4α targets with the hepatic HNF4α expression in patients with advanced liver disease (cohort i).** Spearman´s correlation between the hepatic expression of selected HNF4α target genes and the mRNA levels of HNF4A **(A-C)** as well as the correlation between hepatic mRNA and serum levels of the same markers **(D-F)**. Mouse *L7* (ribosomal) gene was used as an internal control.


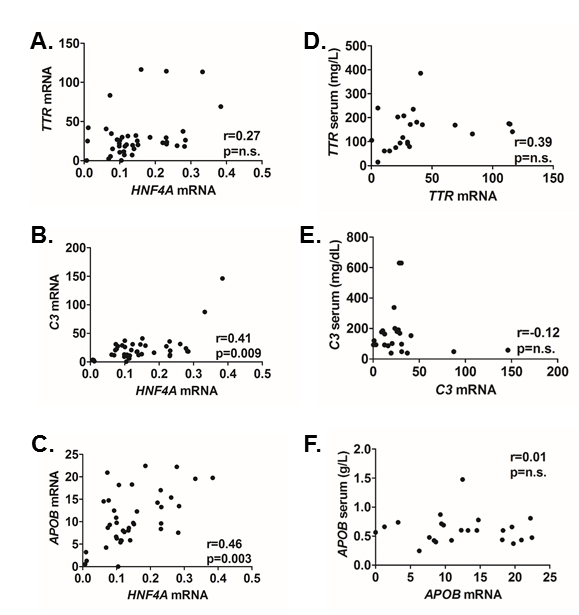


**Additional file 1: Table S1. Primer sequences used for experiments in primary hepatocyte culture.**

| Gene | Primer sequence |
| --- | --- |
| Human Transferrin (*TF*) | F: CGGAAGCCGGTAGATGAATA  R: CCAGATCAAGTCCTCCTTGC |
| Human Hepatocyte Nuclear Factor 4 Alpha (HNF4A) | F: ACTCTCCAAAACCCTCGTCG  R: TCGAGGCACCGTAGTGTTTG |
| Human ribosomal protein (*RPLPO*) | F: GCAATGTTGCCAGTGTCTGT  R: GCCTTGACCTTTTCAGCAAG |
| Human transthyretin (TTR) | F: CAGAAAGGCTGCTGATGACAC  R: ATTCCTCCTCAGTTGTGAGCC |
| Human complement C3 (C3) | F: AAAGAGGACATCCCACCTGC  R: AATGAGGTGCTTCAGCCGTT |
| Human apolipoprotein B (APOB) | F: AGAGCCTACCTCCGCATCTT  R: TTGGAGCCCTTCCTGATGACC |
| Mouse superoxide dismutase 2 (*SOD2*) | F: GAACAATCTCAACGCCACCG  R: GCTGAAGAGCGACCTGAGTT |
| Mouse Transferrin (*TF*) | F: CGCTGGTTGGAACATCCCTA  R: GTGGGCCAATACACAGGTCA |
| Mouse Hepatocyte Nuclear Factor 4 Alpha (*HNF4a*) | F: GGCCAAGATTGACAACCTGC  R: TGAGAGGGCATCGTGTTAGC |
| Mouse nuclear factor of kappa light polypeptide gene (*NFKB1*) | F: TGCTGGAAGTCACATCTG  R: TGCTGAAGGATTCTGTCGTGT |
| Mouse hepcidin antimicrobial peptide (*HAMP*) | F: CTGTCTCCTGCTTCTCCTCCT  R: GGCTGCAGCTCTGTAGTCTGT |
| Mouse ribosomal protein (L7) | F:GAAAGGCAAGGAGGAAGCTCATCT  R: AATCTCAGTGCGGTACATCTGCCT |
